# Early Aseptic Failure of the Tibial Component-Cement Interface in ATTUNE® Total Knee Arthroplasty: A Report of Three Cases

**DOI:** 10.7759/cureus.20582

**Published:** 2021-12-21

**Authors:** John D Murphy, Phillip R Braunlich, William R Judson IV, John N Harker, Patricia A Baumann

**Affiliations:** 1 Orthopaedic Surgery, Largo Medical Center, Largo, USA; 2 Orthopaedic Surgery, C.W. Bill Young Department of Veterans Affairs Medical Center, Saint Petersberg, USA

**Keywords:** attune, aseptic loosening, tibial baseplate, tka, total knee arthroplasty, total knee revision arthroplasty, revision arthroplasty, cement interface, bone cement, painful total knee

## Abstract

Total knee arthroplasty (TKA) systems are designed to maximize the longevity of the implant. However, an unusually high rate of aseptic tibial tray loosening was observed in the DePuy ATTUNE^®^ total knee arthroplasty system (DePuy Synthes, Warsaw, USA). Affected patients noted symptoms of instability and reproducible pain at the bone-implant interface. However, there was no radiographic evidence of lucency or implant failure. Intra-operatively, the tibial component was grossly loose and lacked adhered cement. We hypothesize that the loosening is due to the smooth architecture of the undersurface of the tibial component, lacking either grit blasting or porous coating.

## Introduction

Total knee arthroplasty (TKA) is an important treatment method for addressing refractory knee pain caused by degenerative osteoarthritis. A primary goal of TKA is sustaining the longevity of the implant with minimal chance of the patient requiring a revision arthroplasty. TKA is considered one of the most successful orthopedic procedures, with 12-year survivorship greater than 90% [1]. More so, aseptic failure rates within the first two years are rare, especially when the components are cemented [2,3]. The DePuy ATTUNE® total knee arthroplasty system (DePuy Synthes, Warsaw, USA) was designed with this in mind; unfortunately, its tibial component's smooth under surface design has led to an unusually high early failure rate [1]. The patients in this case report following up within two years complained of anterior knee pain and instability of the knee with ambulation. Radiographic evaluation showed varying degrees of loosening. Intraoperative evaluations found gross loosening of the tibial component, lacking any adherence to the cement. Bonutti et al. described similar findings [1]. The purpose of our study was to expand upon the physical examination and intraoperative findings of patients experiencing loosening of the tibial component of the ATTUNE® TKA, as well as to address treatment options in these patients.

## Case presentation

Case 1

**Figure 1:**
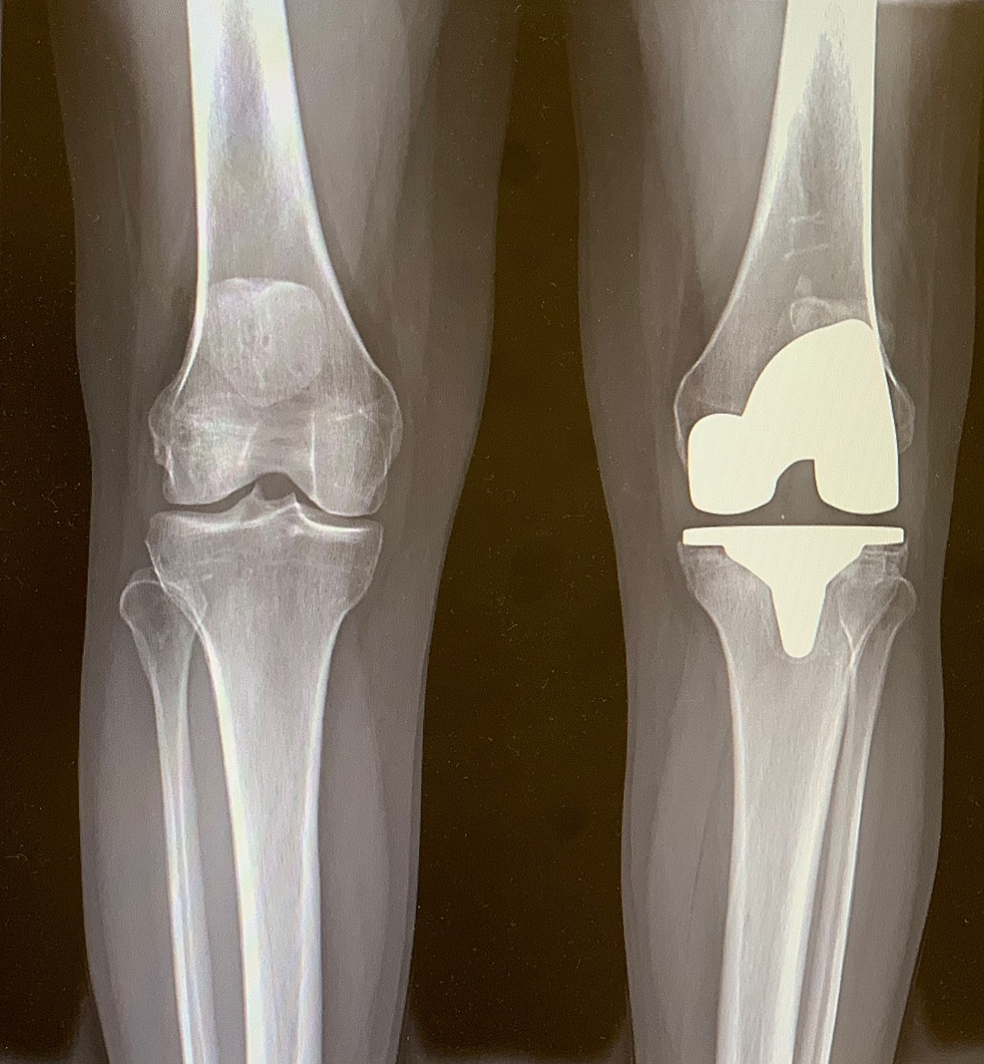
Preoperative standing AP radiograph of the bilateral knees demonstrating no obvious signs of aseptic loosening of the tibial baseplate. AP: anteroposterior

**Figure 2:**
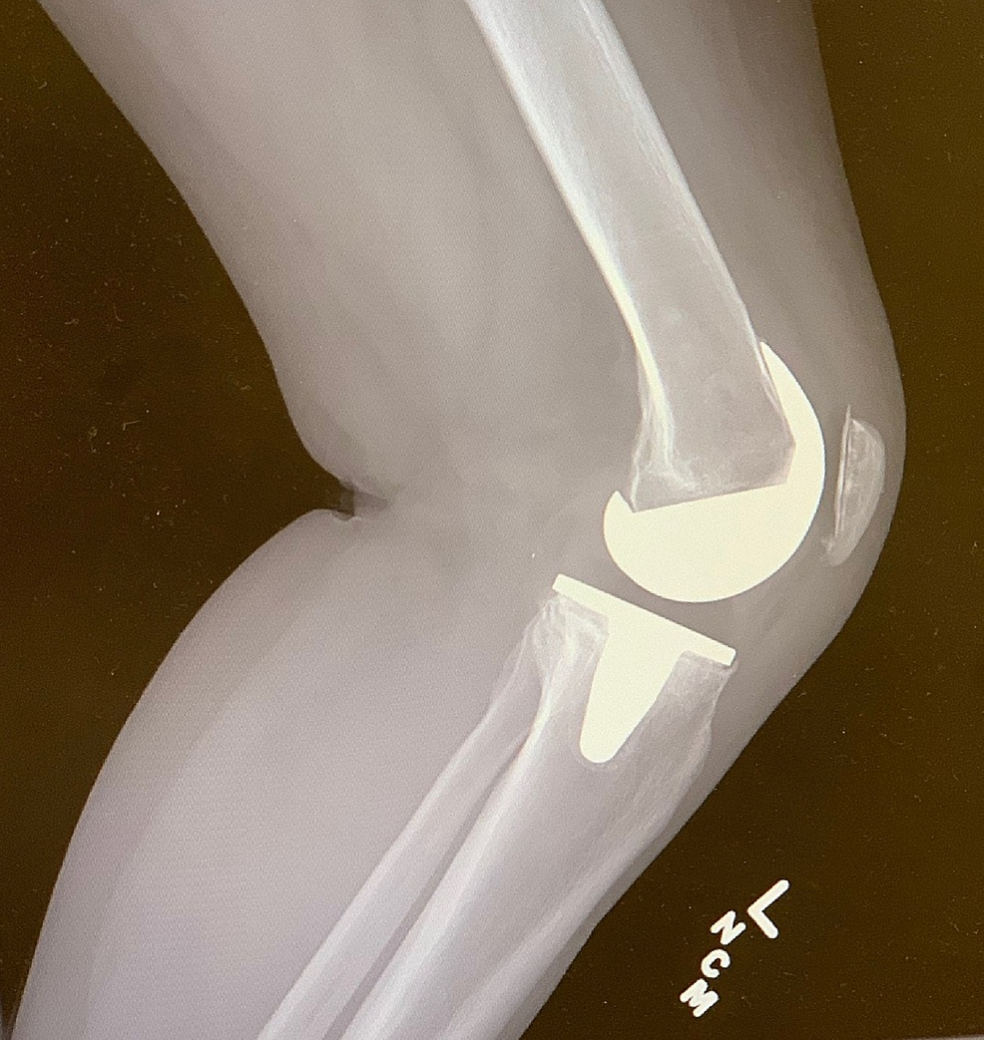
Preoperative lateral radiograph of the left knee demonstrating no obvious signs of aseptic loosening of the components.

A 54-year-old Caucasian female presented to the clinic for a painful left total knee arthroplasty. Her index surgery was performed two years prior with the ATTUNE® TKA system. She endorsed constant pain with weight-bearing and ambulation and admitted to a sensation of instability. She denied fever/chills. Standard workup included ruling out infection with complete blood count (CBC) with differential, erythrocyte sedimentation rate (ESR), C-reactive protein (CRP), all of which were within normal limits. The patient had a well-healed midline incision on physical exam, with no signs of erythema or warmth of the joint. Active range of motion was painless from 0-120° of flexion. There was reproducible tenderness to palpation just inferior to the joint line both medially and laterally at the tibial component-cement interface. There were no signs of gross instability with varus/valgus or flexion/extension stress testing. A review of plain films did not demonstrate any obvious signs of osteolysis around the tibial or femoral components, as seen in Figures 1, 2.

**Figure 3:**
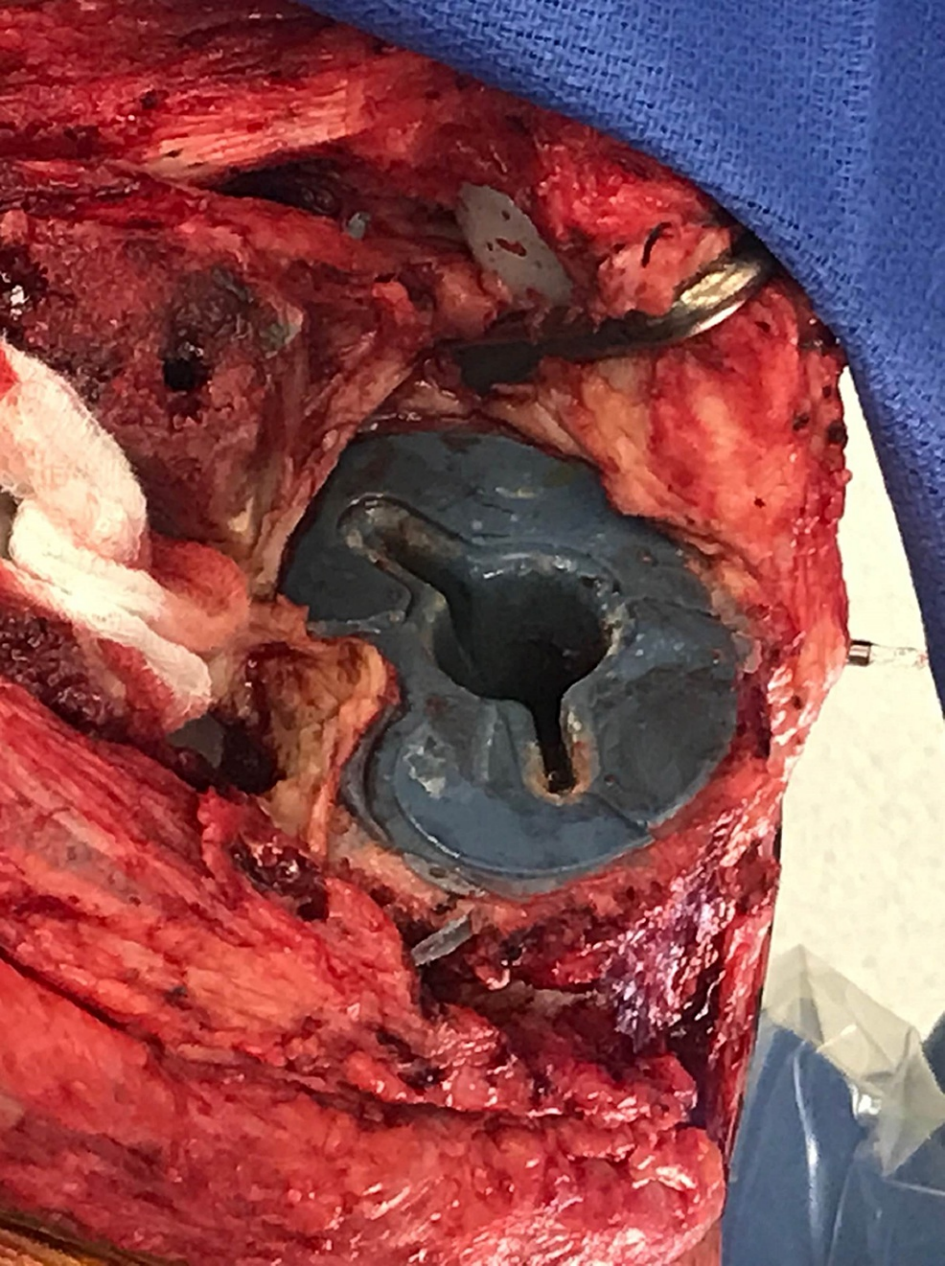
Intraoperative photograph of the left knee demonstrating cement mantel interdigitated to the proximal tibial surface.

**Figure 4:**
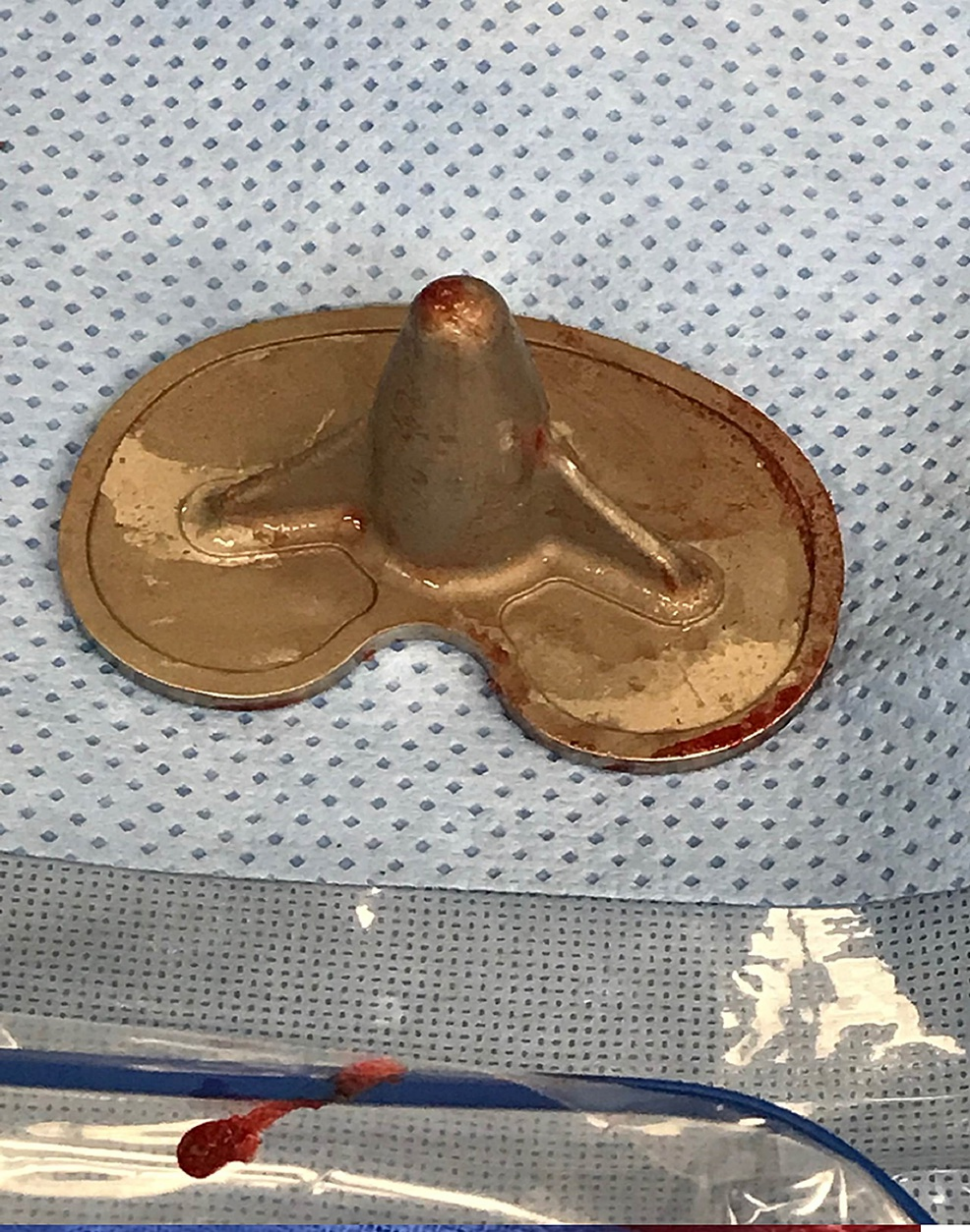
Intraoperative photograph of the undersurface of the tibial baseplate without adhered cement.

Based on the physical examination and familiarity with the early failure of this TKA system, the patient was scheduled for a revision total knee arthroplasty. Intra-operative examination revealed gross loosening of the tibial component. This was tested by the ability to insert the tip of the electrocautery between the cement-tibial component interface. A bone punch was placed under the baseplate to see if the baseplate could be elevated from the cement bone interface. Macromotion was also tested by pressing down on the implant and assessing for movement. Subsequently, an osteotome was used to easily explant the tibial component with a well-maintained cement interface left intact to the proximal tibia (Figure 3). When examining the underside of the tibial component, it completely lacked any adhered cement, as seen in Figure 4. The revision procedure was completed without complication, and the patient had an uneventful postoperative course.

Case 2

**Figure 5:**
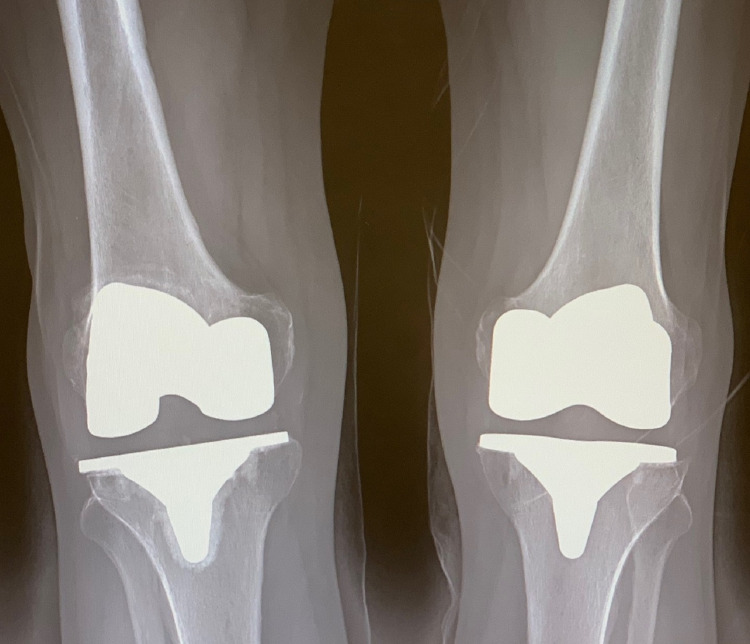
Standing AP preoperative radiograph of the bilateral knees demonstrating aseptic loosening with early mild cystic formation underneath the right tibial baseplate. AP: anteroposterior

**Figure 6:**
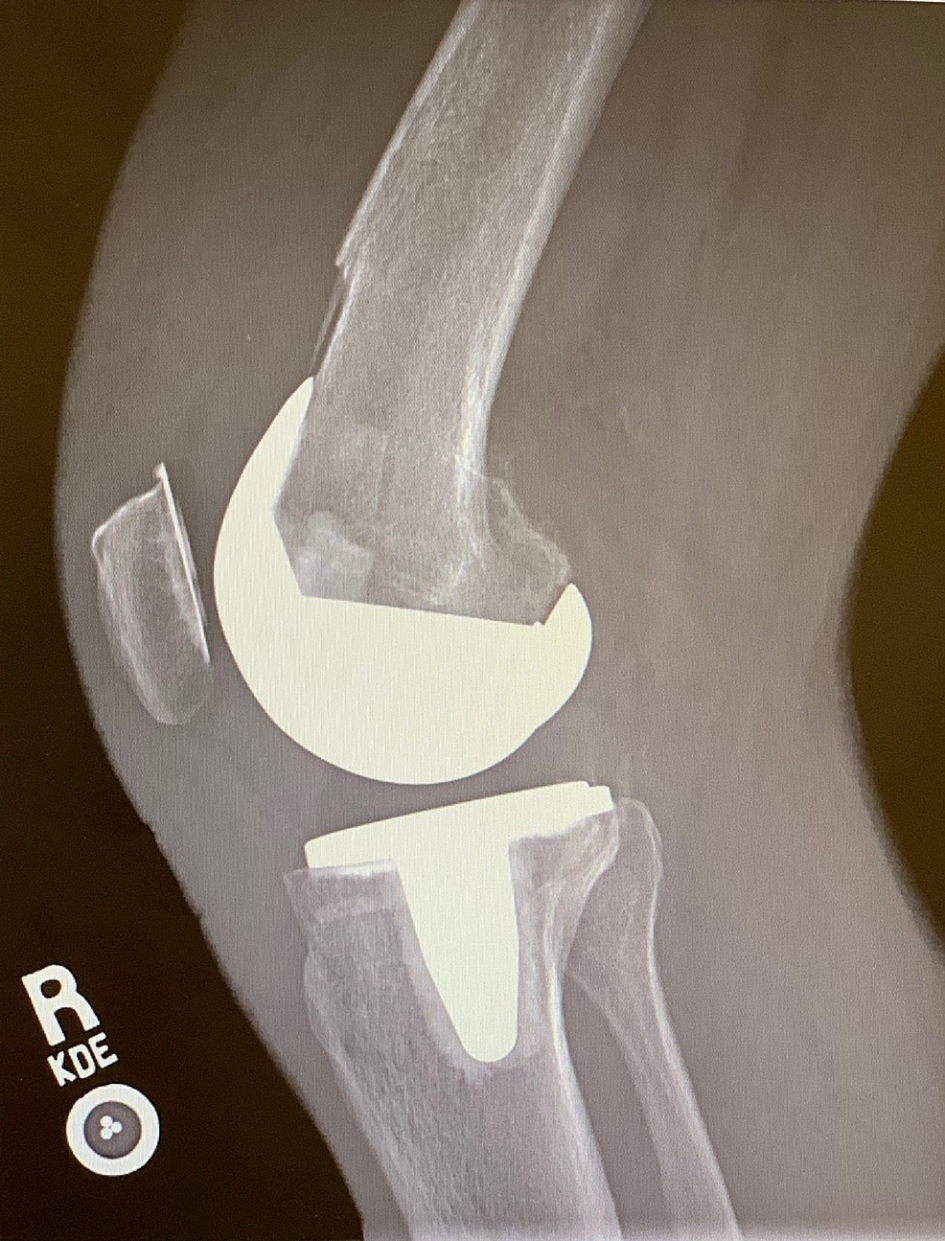
Lateral preoperative radiograph of the right knee demonstrating no obvious signs of aseptic loosening of the components.

**Figure 7:**
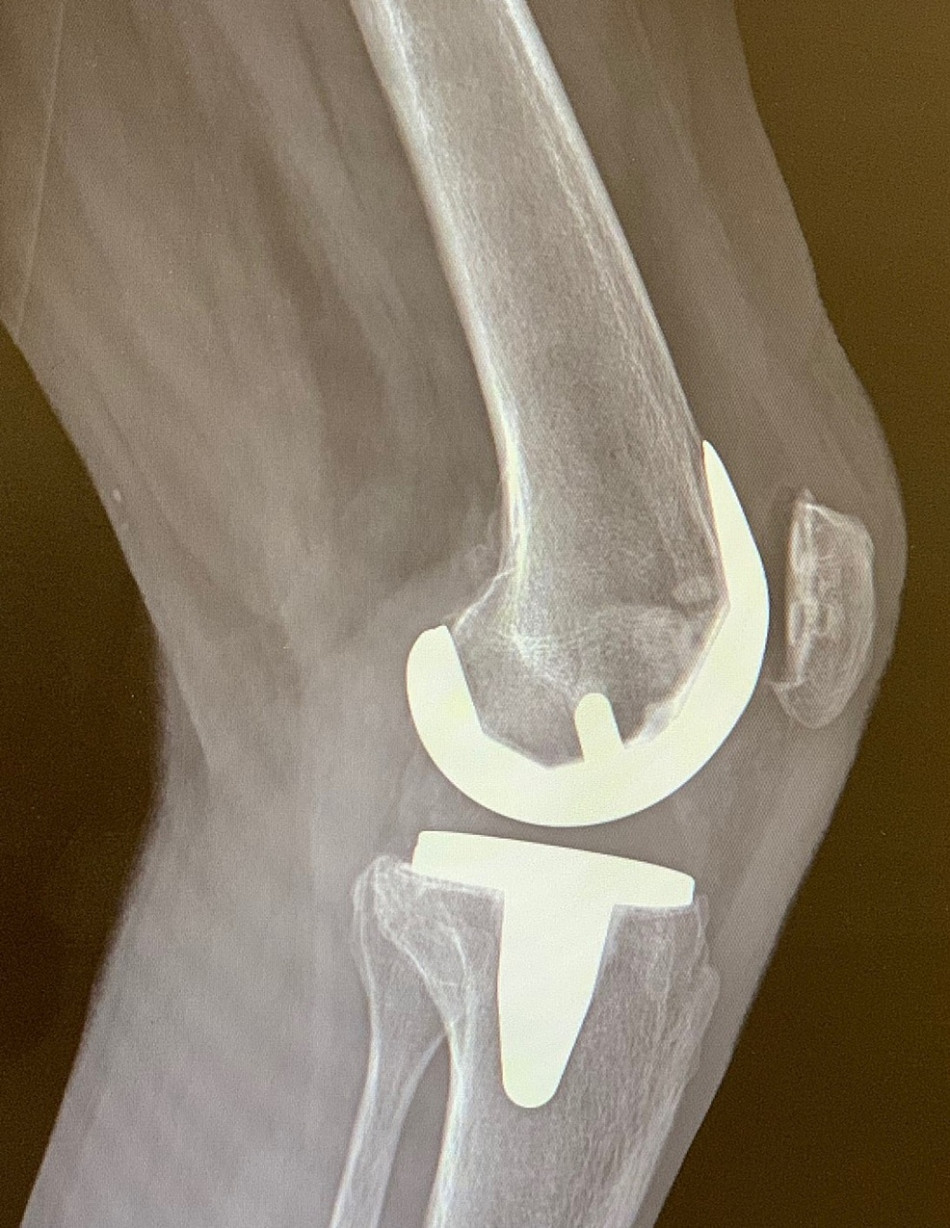
Lateral preoperative radiograph of the left knee demonstrating no obvious signs of aseptic loosening of the components.

A 73-year-old Caucasian male was evaluated at the clinic for a painful bilateral total knee arthroplasty. The left knee arthroplasty was performed utilizing the ATTUNE® TKA system four years earlier, while the right knee arthroplasty was also performed with the same system just three years earlier. He presented with complaints of pain in both knees and a decreased activity level secondary to the pain. He denied any fever or chills, and standard infectious work-up with CBC with differential, ESR, CRP were all negative. On physical exam, the patient had well-healed midline incisions on both knees without signs of infection. Active range of motion of both knees from 0-120°of flexion with pain. The right knee was tender to palpation along the lateral and medial joint lines, with the lateral side being noticeably more tender. The left knee was also tender to palpation medially and laterally. The patient exhibited signs of mild varus/valgus and flexion/extension instability with stress testing. Figures 5-7 are plain film radiographs that demonstrate early signs of osteolysis around the tibial components of both knees with evidence of disrupted cement mantel interface of the right tibial component.

The patient stated his right knee was more painful and was thus consented to a right total knee revision arthroplasty. Intraoperative examination of the tibial baseplate demonstrated macromotion and was easily removed via osteotome and bone tamp. The cement mantel stayed adhered to the bone without evidence of cement adherence to the baseplate. The revision procedure was completed without complication. The patient had an uneventful recovery following the right total knee arthroplasty revision and elected to undergo revision of the left total knee six months later. Once again, intraoperative examination demonstrated a grossly loose tibial component with evidence of macromotion and unadhered cement to the tibial baseplate. Revision surgery was completed without issue, and the patient had an uneventful postoperative recovery.

Case 3

**Figure 8:**
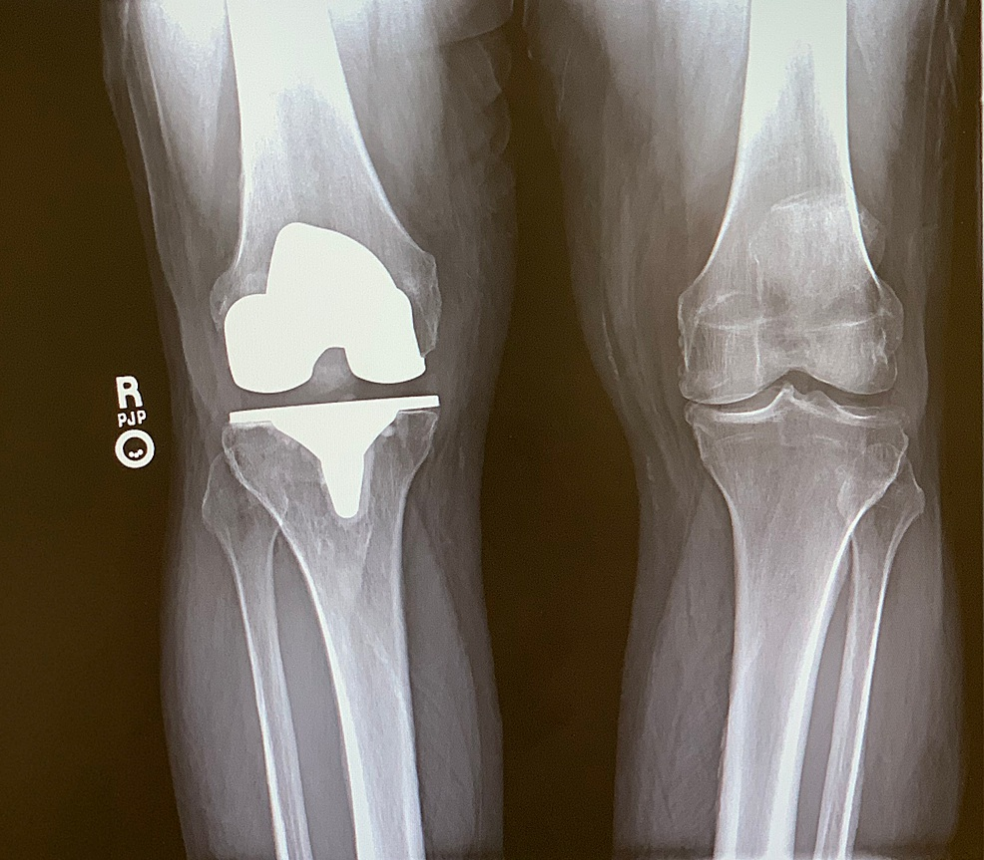
Preoperative standing AP radiographs of the bilateral knees showing lack of cement adherence along the medial aspect of the tibial baseplate with early subchondral osteolysis. AP: anteroposterior

**Figure 9:**
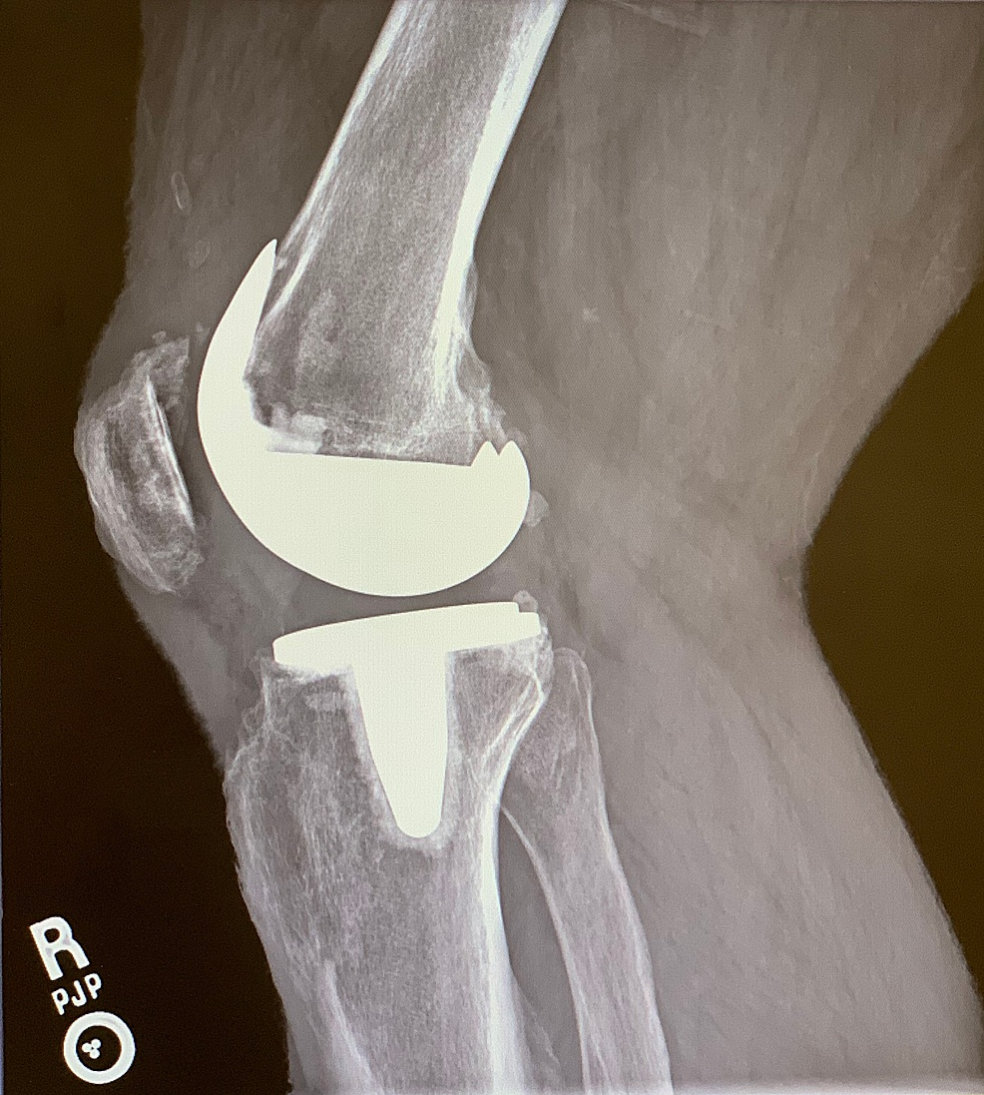
Preoperative lateral radiograph of the right knee demonstrating early anterior subsidence of the tibial baseplate.

A 60-year-old African American male presented to the clinic for a painful right total knee arthroplasty. His primary total knee arthroplasty was performed three years prior with the ATTUNE® TKA system. He complained of persistent right knee pain with weight-bearing and ambulation. He denied any feelings of instability or fever/chills. Infectious workup with CBC with differential, ESR, CRP were all within normal limits. On physical exam of the right knee, the patient had a well-healed midline incision, with no signs of erythema or warmth of the joint. Active range of motion was painless from 0-120°of flexion. Palpation just inferior to the joint line both medially and laterally at the tibial component-cement interface resulted in reproducible tenderness. There were no signs of gross instability with varus/valgus or flexion/extension stress testing. A review of plain films demonstrated obvious disassociation of the medial aspect of the tibial component with subchondral osteolysis (Figures 8, 9).

**Figure 10:**
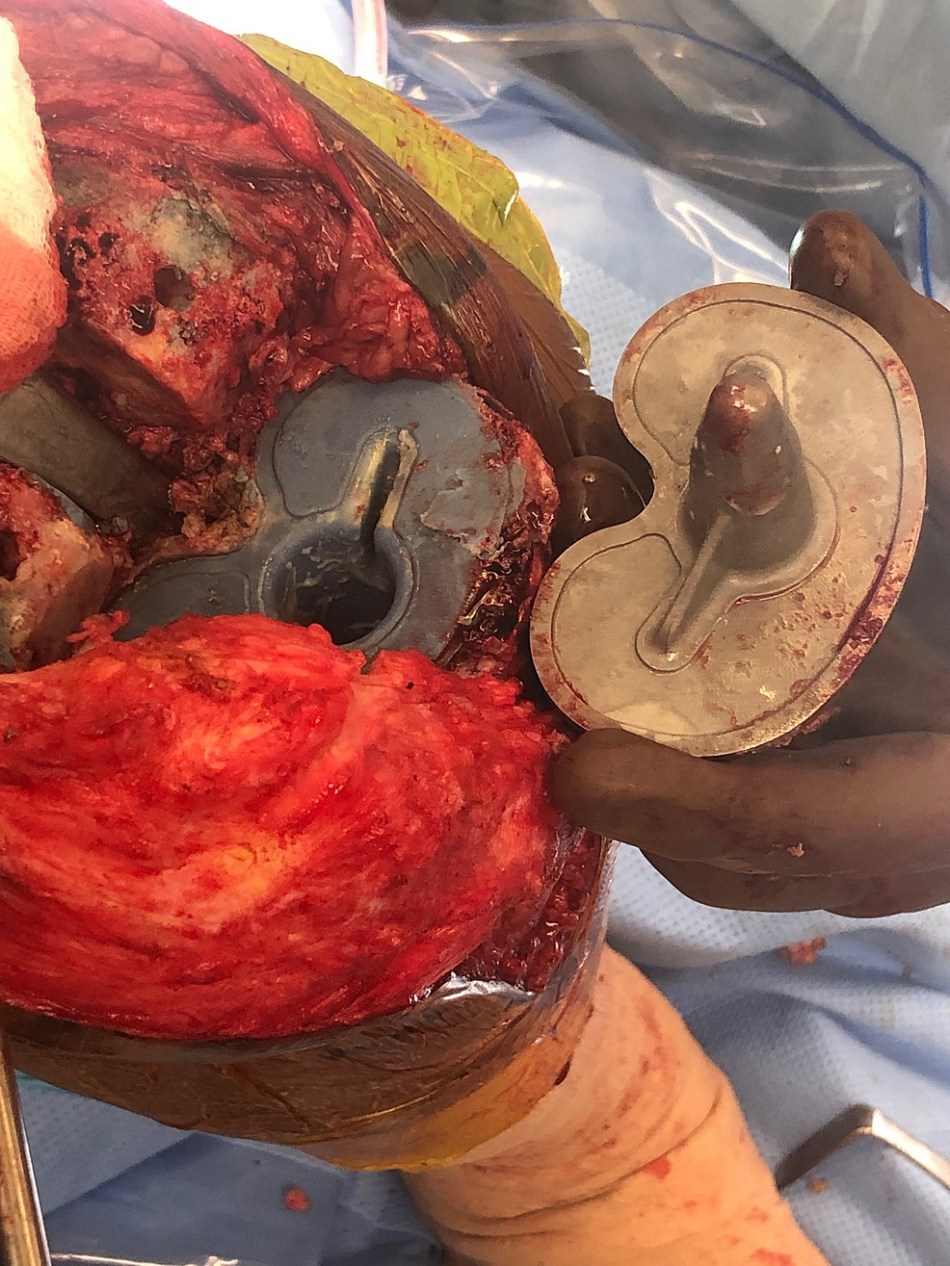
Intraoperative photograph of the right knee demonstrating cement mantel interdigitated to the proximal tibial surface without adherence to the undersurface of the tibial baseplate.

The patient subsequently consented to a right revision total knee arthroplasty. Again, intra-operative examination revealed gross loosening of the tibial component by inserting the electrocautery tip between the cement-tibial component interface. Bone punch was placed on the undersurface of the anterior lip of the tibial baseplate, and a superior force vector was applied by hand, which elevated the baseplate from the tibial cement bone interface. The tibial component was easily explanted with an osteotome, and a well-maintained cement interface remained intact on the proximal aspect of the tibia. The tibial tray was completely lacking adhered cement, as seen in Figure 10. The revision procedure was completed without complication, and the patient had an uneventful postoperative course.

## Discussion

Total knee arthroplasty is designed to reduce pain from end-stage osteoarthritis and provide the patient with excellent survivorship. The ATTUNE® TKA system failed in this regard, with a high propensity of early aseptic failure and loosening. The patients’ complaints of anterior knee pain, a sensation of instability, and the clinical examination of reproducible pain at the tibial component-cement interface led to the decision to treat with a revision TKA. Intra-operatively, the concerns of loosening were confirmed with an easily explanted tibial component and lack of interdigitation of the cement to the baseplate; however, the cement was well interdigitated to the tibia. We feel that the smooth undersurface of the tibial component was a design flaw that led to this problem. The lack of grit-blasting or porous coating did not provide any scaffold for the adherence and interdigitation of the cement to the component. Therefore, the cement adhered well to the proximal tibia but provided no fixation to the tibial component, which allowed small amounts of unintended micromotion with weight-bearing activities. This problem is treated with a revision total knee arthroplasty. In each of our cases, the patients’ instability, anterior knee pain, and pain at the implant-bone interface resolved, and they were satisfied with their outcomes.

Bonutti et al. evaluated reports filed in the Manufacturer and User Facility Device Experience database (MAUDE) about the tibial implant-cement interface [1]. In addition to compiling data from the MAUDE, their study included 15 patients from three hospital databases implanted with the ATTUNE® TKA system experiencing debonding of the tibial implant- cement interface. This study proposed possible etiologies of early aseptic loosening. These include increased constraint, reduced cement pockets, reduced keel rotational stabilizers, and reduced surface roughness of the tibial component compared with the previous generation implant [1]. Regarding the latter, per manufacturer literature, the surface roughness of the tibial component is decreased compared to the femoral component (60 grit blasts vs. 220 grit blasts). Compared to the femoral component, the decreasing grit blast combined with the increased rotational forces and reduced surface area of the tibial baseplate may lead to the failures seen at the tibial implant-bone cement interface [4].

Roughness is not the only factor leading to premature failure of the interface. Cerquiglini et al. examined titanium (Ti) Press-Fit Condylar (PFC) Sigma implants, cobalt-chromium (CoCr) PFC Sigma implants, cobalt-chromium PFC Sigma rotating platform (RP) implants, and ATTUNE implants. This study used a digital imaging method to quantify the amount of cement attached to the backside of each tibial tray. Subsequently, they measured the size of tibial tray thickness, tray projections, peripheral lips, undercuts, and surface roughness (Ra) on the backside and keel of the trays [5]. They demonstrated significant differences in backside surface and stem roughness between the different tibial tray designs (p < 0.0001). Implant designs with the lowest surface roughness, such as Ti and CoCr PFC Sigma designs (Ra median values 0.68 and 0.38, respectively), showed no significant difference in cement adhesion when compared with CoCr PFC Sigma RP, which showed the highest surface roughness (Ra median value 1.95). However, there was a significant difference in cement adhesion compared with the ATTUNE (Ra median value = 1.22 µm). Both the ATTUNE and the PFC Sigma RP lack cement pockets found in the previous generation Sigma implants. When comparing the PFC Sigma RP with the ATTUNE, there is both a peripheral lip with diagonal lips, compared to a single peripheral lip seen with the ATTUNE system, thus decreasing the cement interface surface area. The absence of cement attachment in the ATTUNE design is likely primarily related to the lack of cement pockets seen in previous PFC Sigma designs [5].

Radiographic evidence of failure was appreciated on two of the three patients highlighted in this study. Identification and diagnosis of tibial insert-cement interface loosening cannot be done based on radiographs alone and must be done with patient history and a careful physical exam. Bonutti et al. described radiolucencies on radiographs in only two of 15 patients found to have a failure of the tibial insert-cement interface [1]. In contrast, Yokhana et al. described predictable malalignment with radiolucencies on all 21 TKAs included in their study [2]. The possible lack of radiographic evidence of tibial insert-cement interface loosening compared to other forms of aseptic loosening may lead to an underdiagnosis and thus underreporting this phenomenon.

## Conclusions

Total knee arthroplasty provides successful outcomes and excellent survivorship. However, the DePuy ATTUNE total knee arthroplasty design has led to an unusually high rate of early aseptic failures. Our results were consistent with those of others, concluding the design of the ATTUNE tibial baseplate, with its lack of porous coating or grit blasting, resulted in early implant failure. Without a porous coating or grit blasting, there is no provided architecture for indigitation between the cement and tibial component. This can lead to a persistently loose tibial component, which was allowed small freedom to rotate, causing anterior knee pain and instability. When recognized, this issue is treated with revision total knee arthroplasty.

In our experience, the tibial component-cement interface in patients who underwent total knee arthroplasty with the DePuy ATTUNE system has an unusually high failure rate. Previous literature and our clinical examination and intra-operative observations have demonstrated loosening of the tibial component due to failure at the component-cement interface. Revision total knee arthroplasty is recommended to address pain and instability. Our collection of patients has demonstrated successful outcomes following revision.
